# Supplementary material for: Highly Selective Electrochemical CO2 Reduction to C2 Products on a g-C3N4-Supported Copper-Based Catalyst
Source: Int J Mol Sci. 2022 Nov 19;23(22):14381. doi: 10.3390/ijms232214381 (PMC9696822; doi:10.3390/ijms232214381)
Supplement: Supplementary file 1 [file ijms-23-14381-s001.zip › ijms-2032051-supplementary.pdf]

Supplementary Information

Highly Selective Electrochemical CO_2 Reduction to C_2 Products on a g-C_3N_4-Supported Copper-Based Catalyst

Zijun Yan [1] and Tao Wu [1,2,3,*]

1 New Materials Institute, University of Nottingham Ningbo China, Ningbo 315100, China
2 Key Laboratory of Carbonaceous Wastes Processing and Process Intensification of Zhejiang Province, University of Nottingham Ningbo China, Ningbo 315100, China
3 Nottingham Ningbo Beacons of Excellence Research and Innovation Institute, Ningbo 315000, China
* Correspondence: tao.wu@nottingham.edu.cn

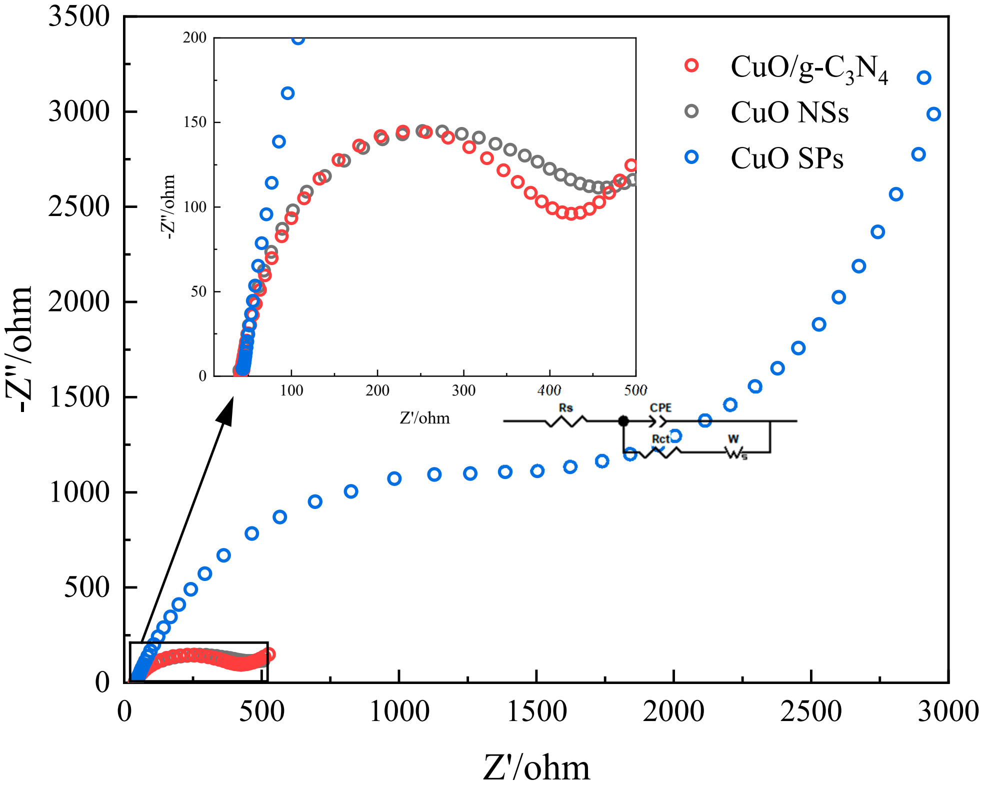

Figure S1. EIS spectra of CuO SPs, CuO NSs and CuO/g-C_3N_4 in CO_2-saturated $KHCO_3$ solution. The enlarged Nyquist plots for the high frequency region and Randel's equivalent circuit model are displayed in the inset.

Table S1. Parameters of EIS equivalent circuit of CuO SPs, CuO NSs and CuO/g-C_3N_4.

Electrode	R_s/(ohm)	R_{ct}/(ohm)	W/(S*$s^{0.5}$)	CPE/(S*s^a)	a
CuO/g-C_3N_4	40.65	361.8	6.539*10^{-3}	101.0*10^{-6}	0.8251
CuO NSs	45.89	702.5	1.169*10^{-3}	70.96*10^{-6}	0.8445
CuO SPs	43.45	1722	0.296*10^{-3}	8.731*10^{-6}	0.8547

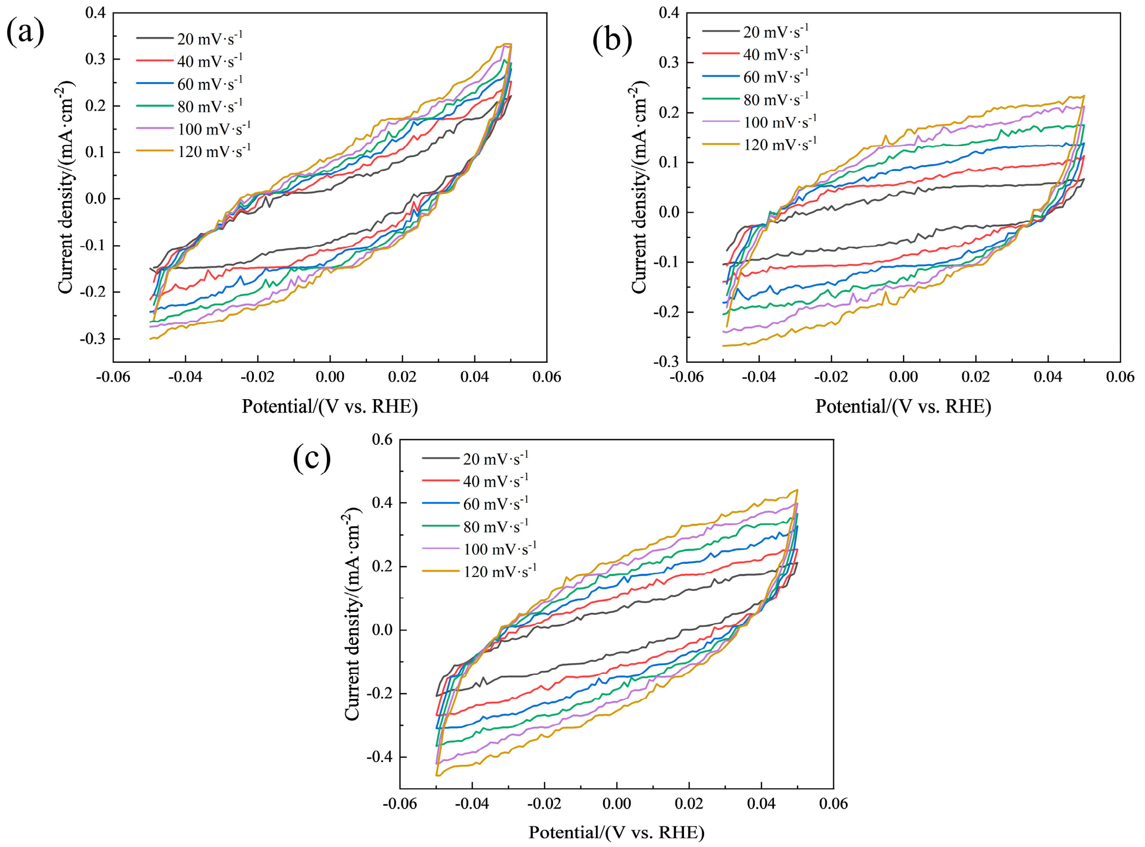

Figure S2. CVs of (a) CuO SPs, (b) CuO NSs and (c) CuO/g-C_3N_4 electrodes at different scan rates (20 ~ 120 mV·s^{-1}) in the potential range of -0.05 ~ 0.05V vs. RHE.

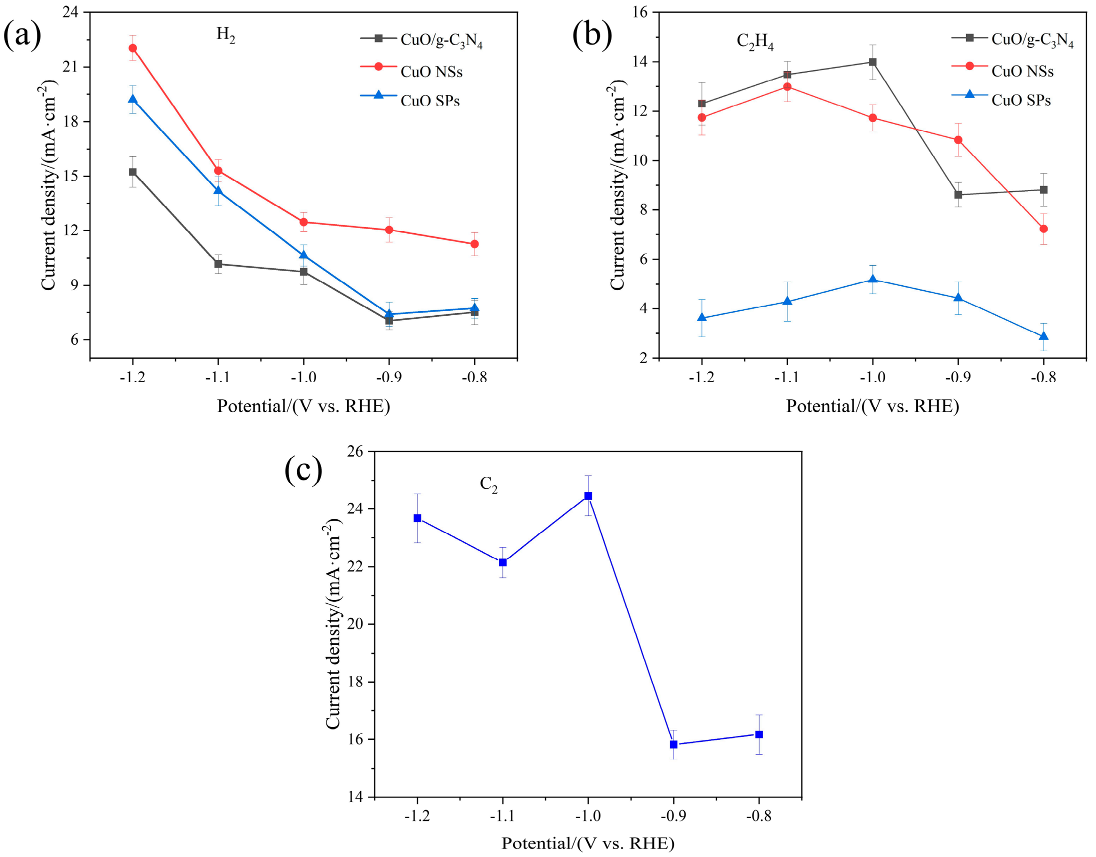

Figure S3. Partial current densities of the products from CO_2 reductions on each catalyst. (a) H_2, (b) C_2H_4 and (c) C_2 for CuO/g-C_3N_4.

Table S2. Comparison of various catalysts for CO_2 electroreduction.

Catalyst	Product	Electrolyte	Potential/(V vs. RHE)	Faradaic efficiency/%	Current density/(mA·cm^{-2})	Ref.
CuO/g-C_3N_4	C_2H_4	0.1 M $KHCO_3$	-1.00	37.0	14.0	This work
	C_2	0.1 M $KHCO_3$	-1.00	64.7	24.5	
Cu NDs	C_2H_4	0.1 M $KHCO_3$	-1.20	22.3	10.0	[1]
HPR-LDH	C_2H_4	0.1 M $KHCO_3$	-1.10	36.0	4.2	[2]
Cu_2O thin films	C_2H_4	0.1 M $KHCO_3$	-0.98	31.0	13.3	[3]
CuZn alloy	C_2H_4	0.1 M $KHCO_3$	-1.10	33.3	2.0	[4]
Prism-Cu	C_2H_4	0.1 M $KHCO_3$	-1.15	30.0	12.0	[5]

Cu_2O-derived Cu	C_2H_4	0.1 M $KHCO_3$	-0.98	43.0	13.3	[3]
CuS_2-Cu-V	C_2 & C_{2+}	0.1 M $KHCO_3$	-0.95	23.0	7.3	[6]
$Cu_2(OH)Cl$	C_2 & C_{2+}	0.1 M $KHCO_3$	-1.20	52.0	31.0	[7]
Cu NPs	C_2 & C_{2+}	0.1 M $KHCO_3$	-0.75	50.0	10.0	[8]
$Cu(Ag\text{-}20)_{20}$	C_2 & C_{2+}	0.1 M $KHCO_3$	-1.10	31.4	7.9	[9]
Cu foil	C_2 & C_{2+}	0.1 M $KHCO_3$	-1.00	60.0	40.0	[10]
Cu_2O-derived particles	C_2 & C_{2+}	0.1 M $KHCO_3$	-1.18	54.4	31.2	[11]
CuX (X = Cl, Br, or I)	C_2 & C_{2+}	0.1 M $KHCO_3$	-1.11	72.6	47.2	[12]

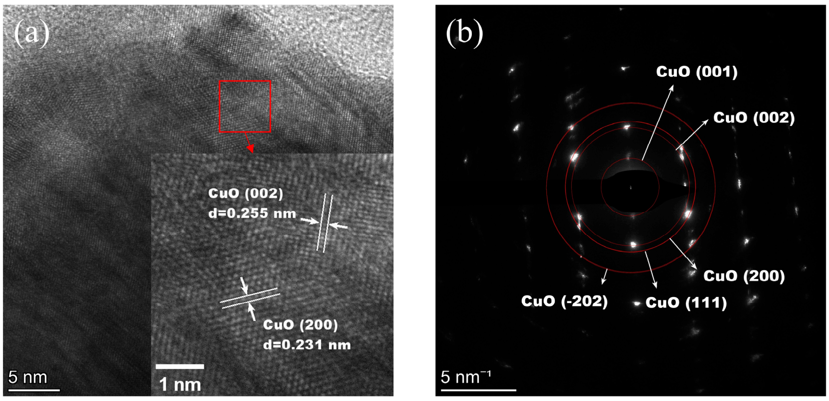

Figure S4. (a) HRTEM and (b) SAED patterns of $CuO/g\text{-}C_3N_4$.

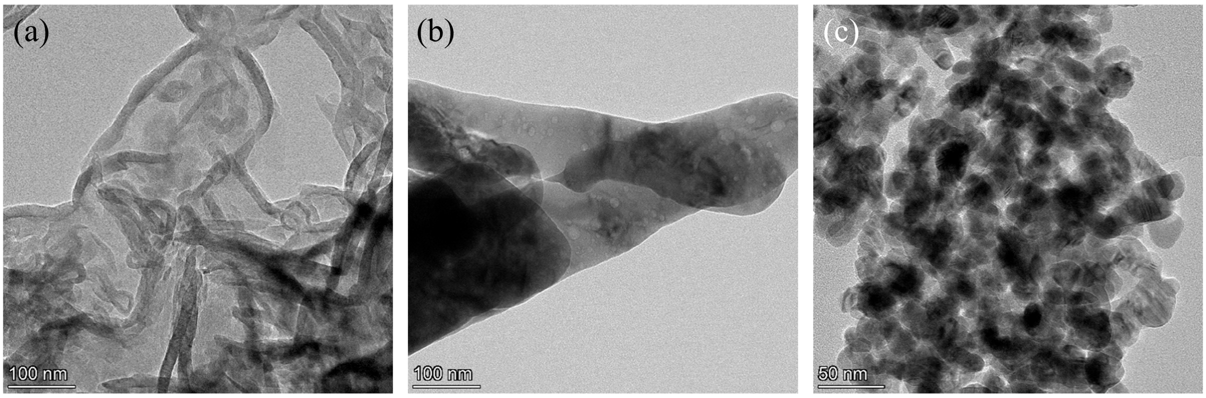

Figure S5. TEM images of (a) $g\text{-}C_3N_4$, (b) CuO NSs, (c) CuO SPs.

References

1. Wu, M.; Zhu, C.; Wang, K.; Li, G.; Dong, X.; Song, Y.; Xue, J.; Chen, W.; Wei, W.; Sun, Y. Promotion of CO_2 Electrochemical Reduction via Cu Nanodendrites. *ACS Appl. Mater. Interfaces* **2020**, *12*, 11562–11569, https://doi.org/10.1021/acsami.9b21153.
2. Altaf, N.; Liang, S.; Huang, L.; Wang, Q. Electro-derived Cu-Cu2O nanocluster from LDH for stable and selective C2 hydrocarbons production from CO_2 electrochemical reduction. *J. Energy Chem.* **2019**, *48*, 169–180, https://doi.org/10.1016/j.jechem.2019.12.013.
3. Handoko, A.D.; Ong, C.W.; Huang, Y.; Lee, Z.G.; Lin, L.; Panetti, G.B.; Yeo, B.S. Mechanistic insights into the selective electroreduction of carbon dioxide to ethylene on Cu_2O-derived copper catalysts. *J. Phys. Chem. C* **2016**, *120*, 20058–20067.
4. Feng, Y.; Li, Z.; Liu, H.; Dong, C.; Wang, J.; Kulinich, S.A.; Du, X.-W. Laser-Prepared CuZn Alloy Catalyst for Selective Electrochemical Reduction of CO_2 to Ethylene. *Langmuir* **2018**, *34*, 13544–13549, https://doi.org/10.1021/acs.langmuir.8b02837.
5. Jeon, H.S.; Kunze, S.; Scholten, F.; Roldan Cuenya, B. Prism-shaped Cu nanocatalysts for electrochemical CO_2 reduction to ethylene. *ACS Catal.* **2018**, *8*, 531–535.
6. Zhuang, T.-T.; Liang, Z.-Q.; Seifitokaldani, A.; Li, Y.; De Luna, P.; Burdyny, T.; Che, F.; Meng, F.; Min, Y.; Quintero-Bermudez, R.; et al. Steering post-C–C coupling selectivity enables high efficiency electroreduction of carbon dioxide to multi-carbon alcohols. *Nat. Catal.* **2018**, *1*, 421–428, https://doi.org/10.1038/s41929-018-0084-7.
7. De Luna, P.; Quintero-Bermudez, R.; Dinh, C.-T.; Ross, M.B.; Bushuyev, O.S.; Todorović, P.; Regier, T.; Kelley, S.O.; Yang, P.; Sargent, E.H. Catalyst electro-redeposition controls morphology and oxidation state for selective carbon dioxide reduction. *Nat. Catal.* **2018**, *1*, 103–110.
8. Kim, D.; Kley, C.S.; Li, Y.; Yang, P. Copper nanoparticle ensembles for selective electroreduction of CO_2 to C2–C3 products. *Proc. Natl. Acad. Sci. USA* **2017**, *114*, 10560–10565.
9. Ting LR, L.; Pique, O.; Lim, S.Y.; Tanhaei, M.; Calle-Vallejo, F.; Yeo, B.S. Enhancing CO_2 electroreduction to ethanol on copper–silver composites by opening an alternative catalytic pathway. *ACS Catal.* **2020**, *10*, 4059–4069.
10. Jiang, K.; Sandberg, R.B.; Akey, A.J.; Liu, X.; Bell, D.C.; Nørskov, J.K.; Chan, K.; Wang, H. Metal ion cycling of Cu foil for selective C–C coupling in electrochemical CO_2 reduction. *Nat. Catal.* **2018**, *1*, 111–119.
11. Torelli, D.A.; Francis, S.A.; Crompton, J.C.; Javier, A.; Thompson, J.R.; Brunschwig, B.S.; Soriaga, M.P.; Lewis, N.S. Nickel–gallium-catalyzed electrochemical reduction of CO_2 to highly reduced products at low overpotentials. *ACS Catal.* **2016**, *6*, 2100–2104.
12. Kim, T.; Palmore, G.T.R. A scalable method for preparing Cu electrocatalysts that convert CO2 into C2+ products. *Nat. Commun.* **2020**, *11*, 1–11.
